# A retrospective and regional approach assessing the genomic diversity of *Salmonella* Dublin

**DOI:** 10.1093/nargab/lqac047

**Published:** 2022-07-09

**Authors:** Madeleine De Sousa Violante, Gaëtan Podeur, Valérie Michel, Laurent Guillier, Nicolas Radomski, Renaud Lailler, Simon Le Hello, François-Xavier Weill, Michel-Yves Mistou, Ludovic Mallet

**Affiliations:** Actalia, 419 route des champs laitiers, CS 50030, 74801 La Roche sur Foron, France; INRAE, MaIAGE, Université Paris-Saclay, F-78352 Jouy-en-Josas, France; Actalia, 419 route des champs laitiers, CS 50030, 74801 La Roche sur Foron, France; Actalia, 419 route des champs laitiers, CS 50030, 74801 La Roche sur Foron, France; ANSES, 14 Rue Pierre et Marie Curie, 94700 Maisons-Alfort, France; Istituto Zooprofilattico Sperimentale dell’Abruzzo e del Molise ‘Giuseppe Caporale’ (IZSAM), via Campo Boario, 64100 Teramo, TE, Italy; ANSES, 14 Rue Pierre et Marie Curie, 94700 Maisons-Alfort, France; UNICAEN, Groupe de Recherche sur l’Adaptation Microbienne, GRAM 2.0, EA2656, University of Caen Normandy, Caen, France; Institut Pasteur, Unité des Bactéries Pathogènes Entériques, Centre National de Référence des Escherichia coli, Shigella et Salmonella, Paris, France; INRAE, MaIAGE, Université Paris-Saclay, F-78352 Jouy-en-Josas, France; Institut Claudius Regaud, 1 avenue Irène Joliot-Curie, 31059 Toulouse Cedex 9, France

## Abstract

From a historically rare serotype, *Salmonella enterica* subsp. *enterica* Dublin slowly became one of the most prevalent *Salmonella* in cattle and raw milk cheese in some regions of France. We present a retrospective genomic analysis of 480 *S*. Dublin isolates to address the context, evolutionary dynamics, local diversity and the genesis processes of regional *S*. Dublin outbreaks events between 2015 and 2017. Samples were clustered and assessed for correlation against metadata including isolation date, isolation matrices, geographical origin and epidemiological hypotheses. Significant findings can be drawn from this work. We found that the geographical distance was a major factor explaining genetic groups in the early stages of the cheese production processes (animals, farms) while down-the-line transformation steps were more likely to host genomic diversity. This supports the hypothesis of a generalised local persistence of strains from animal to finished products, with occasional migration. We also observed that the bacterial surveillance is representative of diversity, while targeted investigations without genomics evidence often included unrelated isolates. Combining both approaches in phylogeography methods allows a better representation of the dynamics, of outbreaks.

## INTRODUCTION

*Salmonella* is one of the most common bacterial pathogens worldwide in human and animal infection (1). The most frequent *Salmonella* subspecies is *Salmonella enterica* subsp. *enterica*, which is one of the four major causes of diarrheal diseases worldwide. Gastroenteritis cases due to Nontyphoidal *Salmonella* were estimated to 153 million annually, including 56 000 deaths (1). Salmonellosis is the second most frequently reported bacteriologically related zoonosis in many European countries (2). The majority of salmonellosis cases cannot be associated with outbreaks and are classified as sporadic cases (3). *Salmonella* exhibits a highly variable host range among animals, especially in mammals and colonizes the gut of various livestock such as poultry, swine or cattle (4). The outcome of infection depends on the pathogenic genotype and *Salmonella* host, ranging from asymptomatic carriage to diverse stages of gastric disorders and in certain cases, evolving into potentially fatal pathogenic conditions.

*Salmonella enterica* subsp. *enterica* serotype Dublin is a host-adapted strain, found especially in cattle farms (5). *S*. Dublin was historically a rare serotype, but slowly became one of the most prevalent *Salmonella* serotype in cattle and cow's raw milk cheese (6,7). Since 2000, *S*. Dublin is often found in the top 20 of most prevalent serotypes at the French National Reference centre for *Escherichia coli*, *Shigella* and *Salmonella* (FNRC-ESS), and has been a persistent cause of human infections for 20 years (8). In France, processes involved in the production of raw milk cheese, uncooked pressed cheese, semi-cooked cheese or soft cheese may prevent the milk to reach a temperature high enough to kill *Salmonella* (9). Beyond the considerable economic losses, contaminated raw milk or finished products infected by carrier cows can cause severe infections in humans (5) and dairy cattle (10). Although diarrhoea is a common consequence of *Salmonella* infections in cattle, the consequences of *S*. Dublin infections commonly reach respiratory syndromes in calves or abortion in gravid cattle (11). *S*. Dublin infections can produce long-term asymptomatic carriers that can periodically shed bacteria in the environment, contributing to the propagation within herds (12), or to humans through direct contact or consumption of contaminated products.

The prevalence of *S*. Dublin in cattle could be explained by a diversity of factors: the bacterial ability to survive in the environment, the symptomatic carriage of individuals, the intermixing of cattle and their exchanges between farms, contaminated food and other factors (13–15). Unfortunately, serotyping or epidemiological data are not sufficient to describe fully contamination links, especially for outbreak events that spans over several months. However, it is possible to trace links between strains at the genomic level which supports hypotheses on the spread, the routes of contamination and history of outbreaks (16).

Diagnostic of *S*. Dublin is commonly based on serotyping (17) and more recently on Whole Genome Sequencing (WGS) methods which have been implemented in many studies and laboratories to improve outbreak resolution (18–20). This method has shown an enhanced cluster detection, an improved resolution and a more accurate result in comparison to laboratory methods (PFGE, MLVA) usually applied to characterize *Salmonella* (21). When performing WGS-based investigations of outbreaks, phylogenomic history is usually reconstructed with core genome point mutations and based on evolution models (22,23). As recently resumed (24), these stochastic point mutations correspond to single nucleotide polymorphisms (SNPs) and small insertions/deletions (INDELs) induced by replication errors or damage of DNA. Point mutation-based phylogenomic reconstructions can be biased when the bacterial genomes are impacted by recombination events (25,26), such as the replacement or inversion of similar sequences (27) (i.e. homologous recombination events), or new genetic material from exogenous genome (i.e. non-homologous recombination events). *S*. Dublin is well known to be impacted by recombination events (28,29) that can induce biases when looking at closely related isolates.

In January 2016, FNRC-ESS, Institut Pasteur, Paris, reported to Santé publique France, the national public health agency, an increase of *Salmonella* Dublin infections across the country. After an epidemiological and microbiological investigation with the help of WGS data, pointed to two types of raw-milk cheeses as vehicles of the *S*. Dublin outbreak at that time (30), a working group was formed to perform a retrospective study focusing on this serotype in the most affected regions. Thanks to the extensive epidemiological and microbiological investigations at the time, a large dataset of strains was collected between 2015 and 2017 in two regions producing the incriminated cheese. The study was designed to understand the circulation of strains and to improve the overall surveillance of *S*. Dublin through whole genome analysis.

We present a retrospective genomic analysis of 480 *S*. Dublin isolates from collections, field investigations, controls and surveillance plans spanning the production process steps from fields to products, all characterised with a set of minimal metadata and contextualised with geographical and temporal links to actual outbreak events. We crossed the phylogenetic signal with metadata to depict and characterize a region-wide *S*. Dublin diversity and strain dynamics over several years to unravel the genesis of outbreak events.

We investigated the impact of homologous recombination events on phylogenomic reconstructions accuracy (i), the correlation of phylogenomic clustering with isolation date, isolation matrices, geographical origin and epidemiological hypotheses (ii), as well as the potential epidemiological relationships with a WGS-based phylogeographic analysis (iii). We highlighted the benefit of WGS approach on closely related isolates and demonstrated how the results from large datasets can help to understand the contamination dynamics of strains, with the objective of supporting sanitary and health authorities to design appropriate safety policies.

## MATERIALS AND METHODS

### Data availability, privacy and anonymization

To comply with statistical, geographical and data privacy, metadata presented in the manuscript were transparently anonymized. Geographical data were anonymized in order to avoid disclosing identity from sparse point distribution, through jittering coordinates with uniform distributions computed separately for longitude and latitude and with amplitudes ranging from −0.2 to +0.2. To improve readability in high-density area, an additional uniform distribution jitter was added, proportional to the point density computed on all coordinates with an amplitude of up to +0.2. All raw sequencing materials used in the manuscript are publicly available (Bioproject: PRJNA737646).

### Collection of samples and related metadata

In this study, four laboratories from the public and private sectors collected 2249 strains of *S*. Dublin between 2015 and 2017 (31). Samples were collected all along the production stages from cows to finished products. After harmonization of metadata, 2101 strains of *S*. Dublin were sub-sampled using the Gower Algorithm (31) in order to build a representative collection of samples as described below. The laboratory for food safety in Maisons-Alfort from ANSES provided 77 samples from its collections of strains, through the French food-chain surveillance (SCA) platform and the biennial ‘National Salmonella Surveillance plan’ 2015 and 2017 (www.plateforme-sca.fr). One sample was collected in December 2014, and was added due to temporality proximity with the dataset.

Targeted samples (selections A, C, D, E) linked to cases of salmonellosis under local epidemiological and microbiological investigations were added in the study. The samples of the selection A are restricted to a single dairy farm where strong clinical signs of salmonellosis cases in cows were repeatedly observed over the years. One sample from 2009 and one sample from 2010 were added to investigate the persistence of the strain within the herd. The samples of the selection C come from a limited geographic area, where contamination from cattle to cheese was found. The samples of the selection D correspond to a period in a restricted geographic area with a large number of milk samples contaminated with *S*. Dublin. Finally, the samples of the selection E were isolated from cattle in a restricted area.

### Strains selection

In order to downsample the dataset of isolates representing the *S*. Dublin diversity, we used a previously described method (31) leveraging available metadata (i.e. year sample, region, production stage and type). Based on Gower coefficient (GC), the distance between two units is the sum of all the variable-specific distances associated to the metadata, whose attributes have a mixed of categorical and numerical values. Each variable can have a weight and consequently change the importance of each metadata class. First, dissimilarity matrix between samples is computed using Gower's distance. Then, hierarchical clustering is applied on the dissimilarity matrix to cluster samples. Finally, the ‘silhouette’ plot is displayed, measuring how close each point in one cluster is to the points in the neighbouring cluster (Supplementary Figure S1-A). The script is available on https://github.com/lguillier/LISTADAPT/tree/master/metadata2assocation. Out of the 2101 samples, 398 samples were drawn from a random selection weighted to balance Gower clusters representation and maximize diversity of sampling (Supplementary Figure S1-B). Finally, 104-targeted samples from selections A, C, D, E were added to the dataset.

### DNA extraction and sequencing

*S*. Dublin strains were isolated and grown on *Salmonella*-selective media (XLD or BHI) and the genomic DNA was extracted using the ‘KingFisherTM Duo Prime’ protocol. Then, the quantity, purity and integrity of DNA samples were assessed using a Qubit, a Nanodrop and electrophoresis migrations on agarose gels, respectively. Next generation sequencing (NGS) was performed by the ‘Institut du Cerveau et de la Moelle Épinière’ (ICM − Hôpital Pitié Salpêtrière, Paris). More precisely, the NGS libraries were prepared using the Nextera XT DNA Library Prep Kit and paired-end sequenced (2 × 150 bases) with an Illumina NextSeq500 sequencer.

### Other studied genomes

Human samples raw reads from a previous study (30) were downloaded from the Sequence Read Archive (SRA) (32) (Bioproject: PRJEB28817).

### Sequence assembly

The assembly was performed with ARTwork, a freely available workflow developed by the team GAMeR at ANSES (https://github.com/afelten-Anses/ARtWORK). In summary, ARTwork estimates the coverage of reads dependently of the LT2 reference genome (bbmap (33)), normalizes the reads (bbnorm (34)), controls the quality of the reads (fastqc (https://github.com/s-andrews/FastQC)) and trims them (35). Then, *de novo* assembly is performed with SPAdes (36), PubMLST scheme is detected by MSLT (https://github.com/tseemann/mlst) and closest reference is retrieved using Mash (37). Finally, scaffolding is performed with Medusa (38), gap filling is done with GapCloser (39), contigs are trimmed with Biopython (40) and an assembly synthesis is carried out with QUAST (41). Three samples displayed sequencing errors and could not be assembled.

### Quality assessment

Quality control was systematically performed and subsequent assemblies failing to meet a set of highly stringent rules were discarded. We rejected samples matching any of the following criteria: more than 1 000 000 assembled bases unaligned to the reference, less than 4 000 000 assembled bases aligned to the reference, >2 INDELs per 100 kb, <80% of assembled bases with 30× coverage, absence of the genome fraction estimation computed by QUAST, or assembly fragmented into >200 contigs. Potential inter- and intra-genus contaminations were detected using Confindr (42) based on assembly metrics and blast respectively. Samples with inter- or intra-genus contamination according to the default Confindr parameters (samples with multiple genera found in the Mash screen step or more than two single nucleotide variants (SNVs) in ribosomal genes) were discarded from the study. Finally, sample serotyping was performed *in silico* based on the assembled genomes using SeqSero (43). Unless conflicting or with reasonable doubt on the error source (metadata, low coverage, etc.), lab-typed and predicted serotypes other than *Salmonella enterica* subsp. *enterica* serotype Dublin have been discarded.

To extend the quality check beyond the sample-specific metrics, we enforced a dataset-wide two-factor (breadth of coverage × depth of coverage) criterion. In a large-dataset context, each genome with its own depth of coverage variation along its sequence exhibits segments with low coverage, thus locally preventing a sound SNP calling. When adding up samples in a dataset, the number of regions where the minimal depth of coverage is not met for at least one of the samples steadily increases. This figure rises sharply when poor quality samples are included in the data set, drastically reducing the breadth of sequence actually used for the phylogeny reconstruction and isolate discrimination. To ensure data consistency and maximize callable SNP positions in a core genome SNPs (cgSNP) analysis context, we rejected high core-losing samples as identified by iteratively comparing depth of coverage drawn from N-samples to that drawn from N-1 samples. Depth of coverage was calculated for each isolate using samtools depth (44). We implemented this method using an in-house filter that keeps each sample containing more than 4.0M positions covered at more than 30×, altogether resulting in a dataset-wide >30×, with a core genome size estimated at 3.8 Mb.

### cgSNP caller and phylogenetic inference

**Figure 1.**
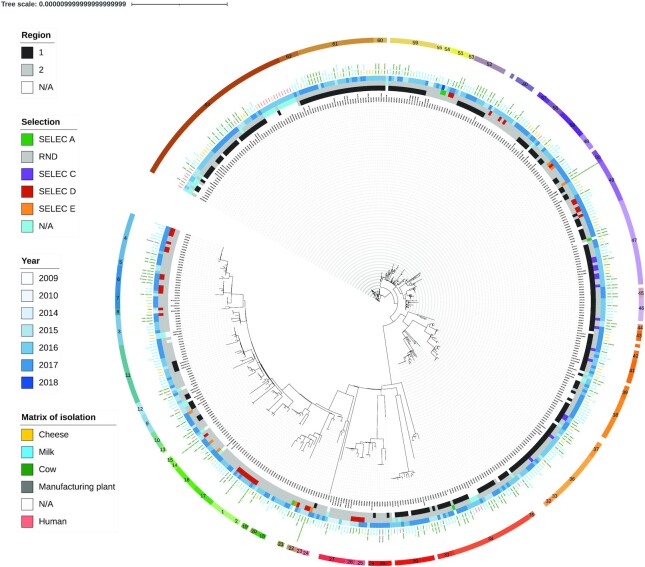
Core genome SNP-based phylogenomic reconstruction by Maximum Likelihood. Reconstruction excluding SNPs from homologous recombination events of *Salmonella* serotype Dublin isolated in two French regions (1 and 2) between 2009 and 2018. Outer ring represents clusters calculated by rPinecone, assigned by numbers and colours. Others rings are represented in the figure and describe isolation year and the isolation matrix. The regions 1 and 2 represent administrative districts in France. The term ‘SELEC A, C, D, E’ represents the four epidemiologic clusters which are investigated. RND is a random selection of samples.

The cgSNP were detected using iVARCall2 (45) which maps (BWA (46)) trimmed reads (Trimmomatic (35)) on the *Salmonella* Dublin CT_02021853 reference, sort reads (Samtools), remove duplicates of mapped reads (Picard (47)), and realign reads around INDELs before detecting variants with HaplotypeCaller from the Genome Analysis ToolKit (GATK) (48). Pseudogenomes have been reconstituted as previously described (45,47). Variants from homologous recombination events were excluded using ClonalFrameML (27). Phylogenetic inferences for both trees were performed by IQ-TREE (49). Core genome SNP-based phylogenomic excluding SNPs from homologous recombination events is unrooted, following an evolutionary model K3Pu + F + I. The consensus tree displayed in Figure 1 was obtained after convergence at 103 iterations with an optimal log-likehood of −6724901.

### Homologous recombination filtering

Recombination tracks were identified using ClonalFrameML (27) with the following parameters set to true: -em, -guess_initial_m, -use_incompatible_sites, -reconstruct_invariant_sites, -output_filtered. The parameter -emsim was set to 20 and other parameters were kept to their default values. Required inputs were constituted by a multiple sequence alignment and a sample tree produced as follow: a reconstructed pseudogenome sequence was generated individually for each sample by mapping the sequencing reads against the *Salmonella* Dublin CT_02021853 reference genome, calling consensus variants and reporting them back onto the reference sequence. Pseudo-sequences from all the samples were piled up to yield the pseudo multiple sequence alignment. IQTree was subsequently used with default parameters on this multiple alignment to build the primary sample phylogenetic tree. Robustness was tested with IQTree parameters -alrt 1000 and -bb 1000. In order to comply with ClonalFrameML, the primary tree was rooted using a midpoint method as implemented in FigTree (https://github.com/rambaut/figtree/). Following evidences sustaining that phylogenetic inferences relying on a Markov chain model of nucleotide substitutions should only take into account points mutations (22). Although filtering of SNPs from homologous recombination events might induce partial loss of information (45,50), we decided to characterize the impact of recombination filtering (Supplementary Figure S2) and subsequently to discuss the results excluding recombinant variants (Figure 1).

### Clustering

rPinecone (51) was used in order to cluster samples, based on a root-to-tip approach with SNP distance relative to ancestral nodes. Given the observed phylogeny, a SNP-scaled tree was generated with pyjar from the rPinecone's main analysis (52), and then a 5 SNP threshold was selected for clustering. A five SNPs threshold is fairly conservative and allows strong assumptions on the links established within clusters, favouring specificity regarding investigated scenarii of reconstruction.

## RESULTS

### Construction of the genomic dataset

After asserting presence of compulsory metadata and harmonizing values, 2 101 samples of *S*. Dublin were sub-sampled using the Gower Algorithm down to 398 (Supplementary Table S1, Random, ‘RND’ throughout the manuscript) (Supplementary Table S1, Gower). Additional samples (*n* = 104) were included to resolve intricate strain detections in unexpected contexts, understand transmission routes and contribute to food production quality standards, 34 of which did not pass filters (Supplementary Table S1, Targeted, containing selections SELEC A + SELEC C + SELEC D + SELEC E). In order to contextualize the regional study with the epidemiological investigation of 2015–2017, paired-reads from Ung *et al.* (30) encompassing samples from food sources and human cases were included in the analysis as well as 77 strains from the ANSES strain collection. Most samples were obtained after 2015, from two regions, and five different matrices: cow, milk, cheese, processing plant and human (Table 1).

**Table 1.** Year, region and matrices of origin of the 480 genomes of *S*. Dublin used in the study

	**Year**		**Region**			**Matrix**				
	≤2015	>2015	1	2	NA	Cow	Milk	Cheese	Processing plant	Human
**Samples**	71	409	256	190	34	167	219	58	5	31

After all the assembly and quality assessment steps, a set of 480 *S*. Dublin genomes associated with trusted samples and metadata was constructed. The set was considered as representative of the diversity of *S*. Dublin circulating in the contaminated area over the years 2015–2017 from both the metadata granularity and the clustering/singleton distribution

### Analysis of the diversity at the core genome level

All samples from the study are predicted as *Salmonella enterica* subsp. *enterica* serotype Dublin by SeqSero (43), and sequence type (ST) 10 by MLST (https://github.com/tseemann/mlst). According to prior knowledge about the clonal expansion of *S*. Dublin (53,54) and within a limited time and geographical span, mutation rate and recombination events were quite low through the genomes of interest. Altogether, 1041 SNPs were detected along the 480 genomes, of which 17 lay within 8 homologous recombination events spanning a total of 299 bp (6 on leaves, 2 on internal nodes). After exclusion of the variants located in homologous recombination segments, 1024 SNPs remained. The core genome SNP-based maximum likelihood (ML) phylogeny topology was slightly impacted by the removal of homologous recombination variants, with only few minor differences observed between nodes (Supplementary Figure S2).

### Phylogenomic reconstruction highlights a regional segregation of *S*. Dublin isolates

The tree excluding the homologous recombination events converged with a commensurate negative likelihood, consistent with the fact that most of the nodes were supported by high bootstrap values (Supplementary Figure S5). Furthermore, the stability of the topology observed upon comparing trees with and without homologous recombination filtering led to establish that the reconstruction based on the cgSNP signal was robust. Two main groups of genomes were defined based on ML inference and confirmed by the sample clustering distance matrix (Supplementary Figure S4), the first one encompassing most of the isolates from the regions 1 (218 out of 272 with region metadata) while the second overall matched region 2 (134 out of 163 with region metadata) (Figure 1, inner circle).

The phylogenomic reconstruction indicated that the isolates do not tend to group by years or matrices. This suggests that within a geographical area there is a continuous exchange of strains between matrices over the years. However, the genomic similarity of a limited number of isolates from different regions indicates that geographical barriers were not completely sealed and that exchanges of strains takes place to some degree between the two regions.

### WGS-based epidemiological investigation and sample clustering

Considering the stringent threshold of pairwise differences defining related genomes (i.e. <5 SNPs), 32 singletons and 63 distinct clusters were defined and encompassed between 2 and 52 samples with a median of 4 (Figure 1). In addition, most of the samples from human cases were clustered within region 1 samples, as previously observed during an investigation performed in 2015 and 2016 by SNP-based analysis and variable-number tandem repeat analysis (MLVA) (30). Finished products related to the human cases were coming from region 1. In the present analysis, we observed that isolates from human cases also clustered with samples from milk, animal and environmental origins. This result shows that strains isolated as part of surveillance plans can provide an early warning of potential future human contamination. The targeted selections (SELEC A, C, D and E) were built on the intuitions of local actors that some isolates might be related. Phylogenomic clustering showed that each of these 4 datasets is polyphyletic. For SELEC D, corresponding to milk samples in a restricted area, the strains were scattered throughout the phylogenetic tree. As a general trend, targeted epidemiological investigations were in every case not in agreement with the genomic evidence. On one hand, some of the genomes were not clustered together, and on the other hand, samples originally considered as not related to any outbreak events were clustered with outbreak strains (i.e. identified as genetically very close to defined genetic clusters) (Figure 1).

### Geolocation and regional segregation of genomic diversity

**Figure 2.**
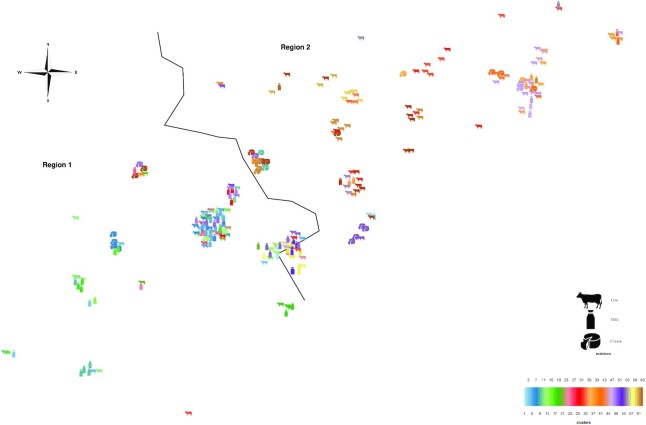
Jitterized geolocation of samples source and genomic clustering of *Salmonella* serotype Dublin isolated in two French regions between 2009 and 2018 (*n* = 261). The border between the two region is coarsely represented by the black delimitation. The clusters were defined with rPinecone set to target 5 or fewer SNP differences within clusters (coloured clusters). Clustering and colour scheme match those on Figure 1. Geographic location has been anonymized by adding a random variable to geographical coordinates. Pictogram of each point describes the matrix from which each isolate was sampled.

In order to investigate in-depth the regional segregation, we collected accurate geographic data (*n* = 261) and built a phylogeography map of the two regions (Figure 2) according to our phylogenomic clustering (Figure 1). This phylogeographic reconstruction suggests that the geographical distance is a major factor in genomic divergence and relatedness for the early stages of the production processes (i.e. animals, farms), while downstream transformation steps are more likely to harbour genomic diversity. Some areas contain different clusters of genomes, especially the areas near frontiers. This observation is likely to reflect the diversity of origin of the samples transferred in these product hubs but also suggests that cross-contamination can occur in these locations. Most of the clusters are geographically packed, including over time, showing a persistence of *S*. Dublin sub-lineages in specific areas. The geolocation also illustrates the diversity of sources associated with most clusters (Figure 2), demonstrating the very local circulation of *S*. Dublin from animal to finished products. For example, the cluster 47 included 11 samples from bovine, 8 from milk and 7 from cheese with some strains isolated from the three matrices being virtually identical (0 SNP difference). Another example is the cluster 61 encompassing 9 samples from cattle, 9 from milk and 5 from cheese. These findings emphasize the persistence of *S*. Dublin along the production chain with highly conserved genomic characteristics.

## DISCUSSION

This retrospective study demonstrates here that continuous genomic surveillance brings valuable information to understand routes of contamination and target sources of contamination faster. Both are key features bolstering investigations in an emergency context. Firstly, the sub-sampling of the strain panel was performed while balancing metadata modality and maximizing diversity representation. Singleton cluster analysis suggested that the sub-sampling covered a large fraction of the diversity and variability present in the available panel of isolates. Moreover, this considerably reduced the cost of sequencing and the computational time of bioinformatics pipelines, installing the genomic surveillance as an economically and timely efficient tool for food safety. Secondly, the core genome investigations together with epidemiological data were found resolutive and robust, allowing an easy and accurate identification of strain links at the regional scale. Finally, *S*. Dublin exhibited genetic diversity specific to its geography, which resulted in local clusters that sometimes intermix through exchange zones.

### WGS analysis brings more insight into outbreak investigations

Investigations on targeted sample selections showed that epidemiological data is not enough to decipher the link between samples. This is particularly true when dealing with closely related strains, in areas foregrounding regular trading and exchanges of food, products and animals linked to the carriage and transmission of *Salmonella*. Thanks to this core genome SNPs, phylogenetic reconstruction highlighted links between strains, which were not identified from epidemiological data, revealing new potential sources of contamination. It was previously shown that WGS can provide more insight in outbreaks investigations (55), thus some public health agencies have developed WGS methods to overcome the lack of precision of *Salmonella* typing methods (19,56,57). In France, WGS is not systematically implemented as the main typing tool for Salmonella in foodborne outbreak investigation. It is therefore difficult to trace back outbreaks. Combined with epidemiological data, investigators can track back the dissemination of strains at the regional scale, and point-out exchanges of strains between places or the origin of their contamination.

### Even few mutations show regional segregation of *S*. Dublin

We discovered a regional segregation of *S*. Dublin in France, which was not previously demonstrated. As previously shown (54,58), *S*. Dublin is a highly clonal serotype and harbours a highly conserved genome (13,53,54,59), which is found here with a low number of intra and inter-cluster SNPs, and from the few numbers of SNPs excluded from recombination events. This result is supported by previously published studies, which revealed low SNP differences between linked isolates and unlinked isolates from the same country (53,60,61). Even if the core-genome SNP pipelines used in these studies are different (62), the orders of magnitude between pairwise SNP differences are similar. More precisely, in the study (53) where samples were isolated between 1996 and 2016, the majority of isolates from the same geographic area clustered with a threshold of less than 10 SNPs. A comparison between French and Danish samples shows a clustering of strains by country (Supplementary Figure S3).

The investigations of the outbreak from French cheeses at a similar period (30) defined clusters and subclusters harbouring less than 15 and 5 SNP differences, respectively. In the present study, we have decided to apply a smaller threshold (five SNPs), given the shorter period of time and the small geographical area considered. A threshold of five SNPs is very conservative and allows identification of related samples. In addition, the drastic curation and quality assessment performed during sample selection in the present study make highly unlikely the detection of erroneous SNPs and recombination. It has been proven that the geographical partitioning impacts the core genome of *S*. Dublin (54), which supports our conclusion that strains considered to be related (i.e. that differ by five or less SNPs) belong to the same geographical area.

### Geographical clusters of *S*. Dublin genomes highlight trading areas

Using the five SNPs threshold, samples were not clustered together by years or isolation matrices. On the contrary, the strains tend to cluster by geographic area, from cattle to finished products, supporting the hypothesis of persistence of the same strains infecting herds and production environments over the longer term. *S*. Dublin can be widespread in the environment and becomes an important source of infection for animals (7), which may have become latent carriers. The hypotheses about potential vertical transmission through carriers (14) would support the geographical segregation of *S*. Dublin observed in the present study. The spread of the strains in these areas could be through the purchase and contact of infected livestock. It is also possible that in these highland areas, the spread could occur through the watersheds and rivers during the rainy season.

Some geographic areas, such as cities located near the border between the two regions, correspond to towns of exchange and gathering of cattle. In this context, animals are exposed to multiple infection risk factors, such as cattle sales or agricultural forums, which promote inter-animal contamination and increase fecal-oral cycle of infection (13). In these cities, we found different strains, which clustered with different regions, showing the magnitude of bacterial exchanges taking place in the area. The region's livestock transport network and the network of farms where cheeses workshops are supplied seem also to harbour a high diversity of *S*. Dublin. Nevertheless, monitoring and investigating these networks to understand the circulation of strains remains difficult due to the amount of data and the patchy nature of sampling.

### *S*. Dublin outbreaks had multiple origins

During the present study, we first analysed a sample set without isolates from human cases. We observed that adding samples from human cases did not change the tree topology. We also noticed that human samples are located in different clusters, showing different contamination origins as shown in Ung *et al.* (30). 4 samples from human cases are singleton, meaning that their origin remains elusive at the time. This observation suggests that the surveillance plan, despite its size and meshing, does not fully cover the diversity that exists in these two regions or that our subsampling failed to represent rare clusters from which those cases arose. The granularity of the surveillance should however not be held as a sole source of data scarcity as a large part of *Salmonella* cases are undetected or unreported (2). Although these four *S*. Dublin are included in the regional phylogenetic tree, the hypothesis of a foreign origin cannot be excluded, as genomic variability has not been studied throughout the country. The paucity of *S*. Dublin cases with available genomic resources and usable geographic metadata prevented deeper investigations.

### Limits and perspectives

The surveillance, despite its size and meshing, does not seem to fully cover the diversity of these two regions up to this SNP resolution, or our subsampling failed to represent rare clusters, as singletons appear in the clustering. The sheer performance of the sampling and subsampling can nonetheless be tallied, as only 32 singletons were found out of 480 samples (6.7%) under the most stringent clustering threshold applied on *S*. Dublin. To answer those hypotheses with an in-depth analysis, we would recommend improving the monitoring plans in view of missing or ineligible metadata related to 219 samples in the present study. These observations emphasize that the implementation of a metadata nomenclature and minimal metadata sets is required for surveillance activities.

After an outbreak event in a cattle farm, all the organic material is removed and the surfaces are washed with water and detergent. A disinfectant is subsequently applied depending on the *Salmonella* species (63,64). After cleaning, measures should be taken to prevent reintroduction of the bacteria. In this study, we have a strong assumption of contamination by contact between animals from distinct facilities or through persistence within the hosts or in the environment. Indeed, heifers, calves and cows infected around the time of calving are the animals with the higher risk of becoming *S*. Dublin carriers (12,65), and environmental contamination from infected calves also plays an important role in the spread of the bacteria within calves (6). To prevent these risks of contamination and infection, biosecurity measures can be proposed. For instance, good calving area management has been associated with the probability of successful control of *Salmonella* (66). Measures, like separating calf pens by solid walls, preventing cows from calving before being moved to the designated calving pen or quarantine newly arrived animals has been proven to be effective against the spread of *Salmonella* in herds. In addition, calves are more likely to be seropositive in farms, thus monitoring the serology of all calves can predict a new outbreak within the herds (67). It could be recommended that on-farm hygiene measures be increased to limit the likelihood of transmission to cows during the production period and that milking hygiene measures be reinforced to prevent contamination of milk. Finally, milk from farms suspected of having active circulation of S. Dublin on the farm could be temporarily excluded from the production of raw milk cheeses

## CONCLUSION

In this study, epidemiological and genomic data allowed the characterization of the diversity and understanding of phylogeographic location of *S*. Dublin strains. The precision brought by WGS methods bolstered the identification of different clusters and uncovered links between samples.

Our results display the geographical distance as a major factor in genomic divergence and relatedness for the early stages of the production processes (animals, farms), while down-the-line transformation steps are more likely related to host genomic diversity. The discriminative signal between samples from region 1 and region 2 from their genetic content is a precious result that can be used in the future to track back contaminations. These findings are in favour of a generalised persistence of local strains and occasional migration with a strong phylogeographic context. These findings also suggest that *S*. Dublin in those regions are geographically segregated with clusters containing different matrices potentially emphasizing spreading the bacteria over the entire food chain, and within herds. Geographic locations showing a high diversity of *Salmonella* were found to be exchange areas with several cooperatives or a large concentration of markets where different bacteria from different geographical locations meet each other. Control measures must be put in place in these exchange areas to prevent the spread of different clusters of *Salmonella* found in humans.

We appraise the benefit of a WGS cgSNP approach on closely related isolates and how the results from large datasets under proper control of the impact of breadth-of-core erosion can bring to fathom strain contamination dynamics and empower sanitary and safety authorities in designing tailored safety policies.

Altogether, the present results brought an insight on regional genomic diversity of highly related genomes involved in foodborne outbreaks, underlining the necessity to drive investigations toward the most resolutive comparative genomics methods. These findings pave the way toward the development of news comparative tools integrating others sources of variation as a discriminative metrics along with SNPs, such as INDELs, structural variations, mobilome and accessory genome contingency.

## DATA AVAILABILITY

The core genome calculation of positions covered at more than 30X is available at: github/madeleinevlt. The figures and scripts are available at: github/madeleinevlt.

Raw reads of the project have been deposited in SRA under accession number PRJNA737646.

## Supplementary Material

lqac047_Supplemental_Files
