# Supplementary material for: A retrospective and regional approach assessing the genomic diversity of Salmonella Dublin
Source: NAR Genom Bioinform. 2022 Jul 9;4(3):lqac047. doi: 10.1093/nargab/lqac047 (PMC9270687; doi:10.1093/nargab/lqac047)
Supplement: lqac047_Supplemental_Files [file lqac047_supplemental_files.zip › Supplementary_legends.docx]

Supplementary Table S1: Sheet 1: metadata of the 480 *S*.Dublin genomes used in this study : sample ID and name, collection year, geographical origin. Sheet 2: Metadata used in Figure 2. Sheet 3: Genomes quality.

Supplementary Figure S1: Gower’s results of the 398 randomly selected panel. A : Gower’s distance agglomerative clustering. X axis represents the number of clusters, Y axis represents the average silhouette width. B : Dendrogram plot of Gower’s distance for cluster 30. Samples are coloured by clusters.

Supplementary Figure S2: Impact of homologous recombination events on phylogenetic topology. Left: Phylogenetic tree with recombination events. Right: Phylogenetic tree with recombination events detected by ClonalFrameML and excluded.

Supplementary Figure S3: Comparison of 43 *S*. Dublin samples from France (in blue) and Denmark (in red). Phylogenetic tree is made by IQTREE with an evolutionary model K3Pu+F+I model and an optimal log-likelihood of -6728504.9209.

Supplementary Figure S4: Heatmap of intra and inter distance of clusters selected by rPinecone. Distances are represented in SNP, from low value (blue) to high value (yellow).

Supplementary Figure S5: Coregenome SNP-based phylogenomic reconstruction by Maximum Likelihood with bootstrap values. Only bootstrap values greater than 80 are displayed.
