# Supplementary figures and images for: A retrospective and regional approach assessing the genomic diversity of Salmonella Dublin
Source: NAR Genom Bioinform. 2022 Jul 9;4(3):lqac047. doi: 10.1093/nargab/lqac047 (PMC9270687; doi:10.1093/nargab/lqac047)

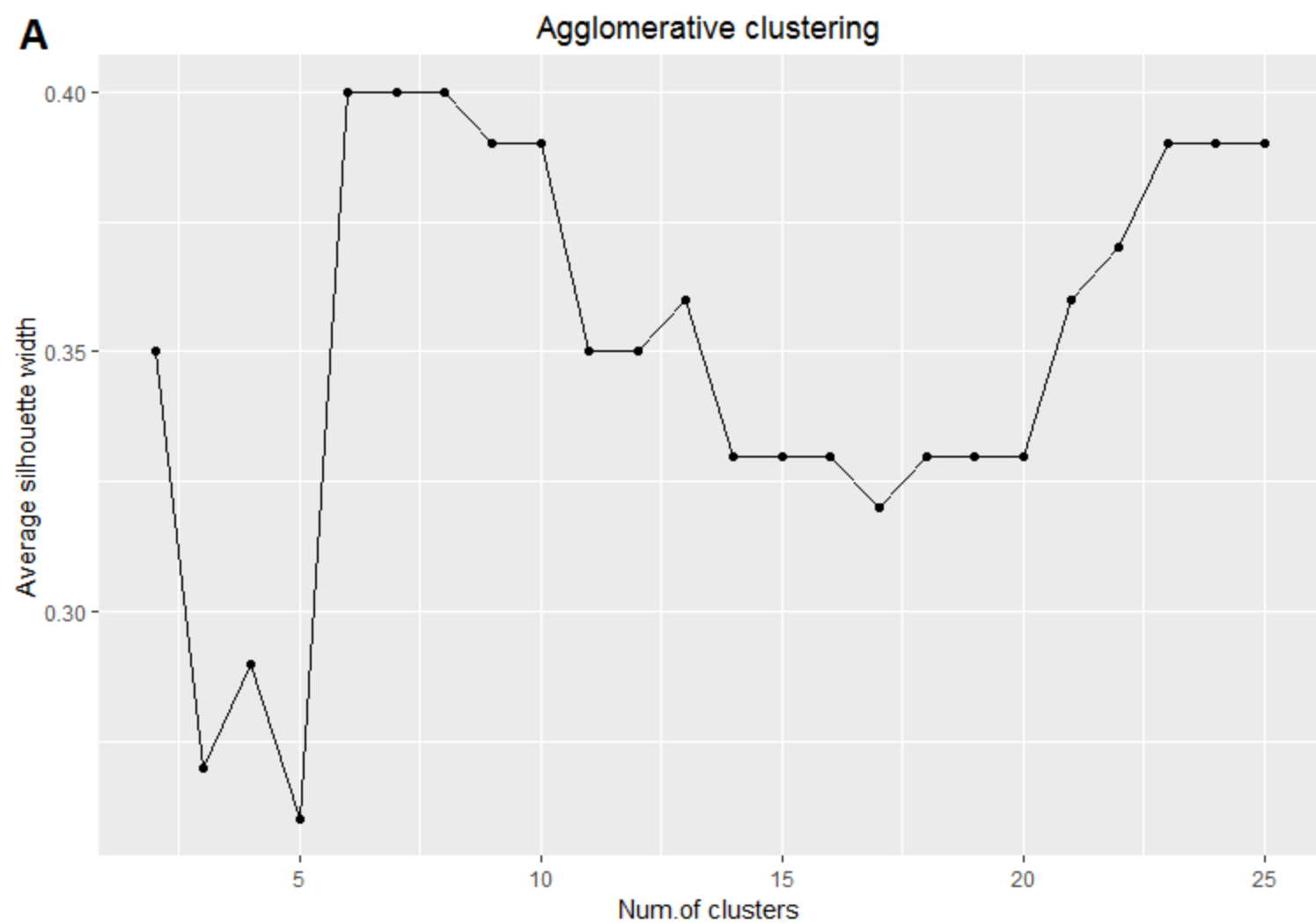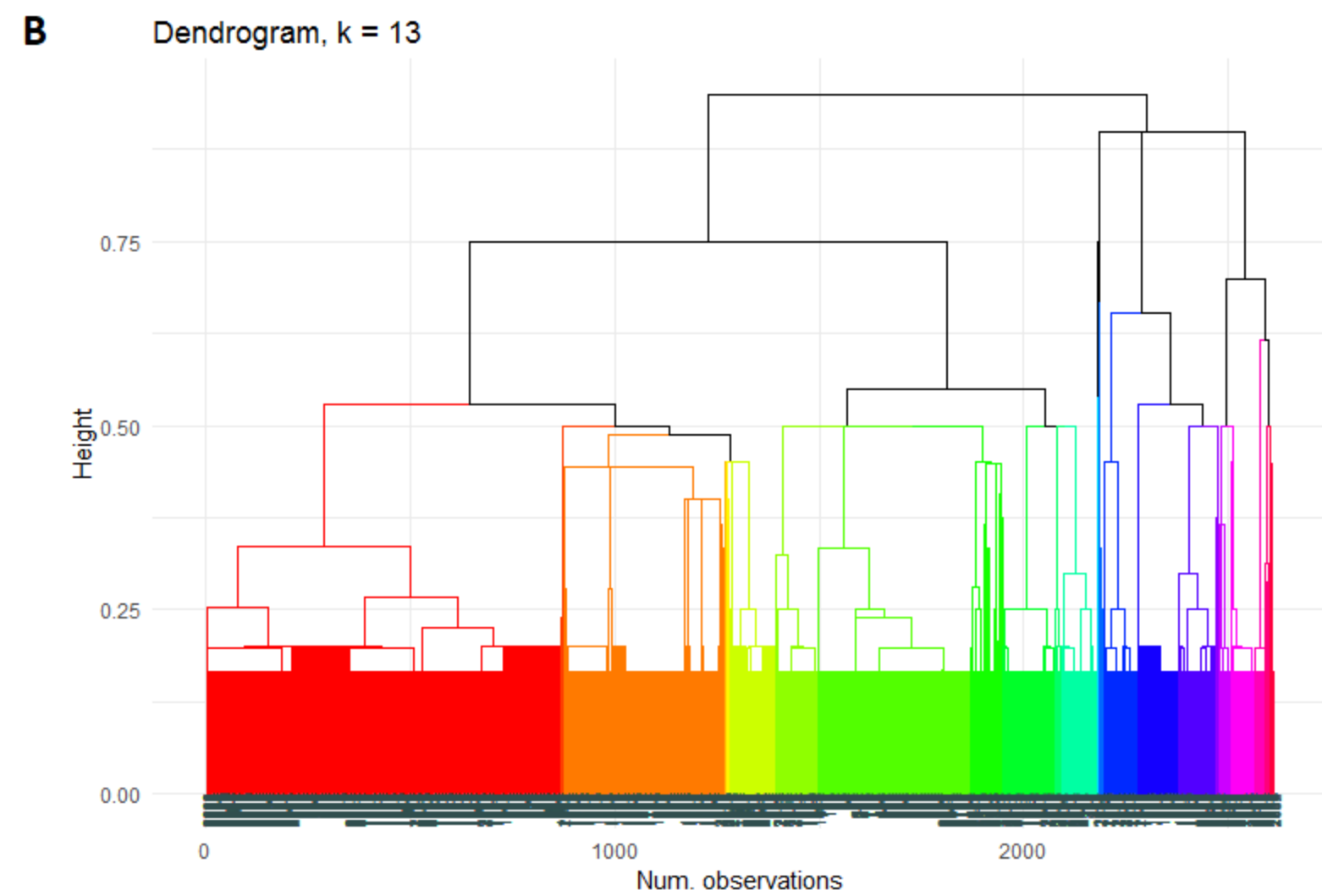

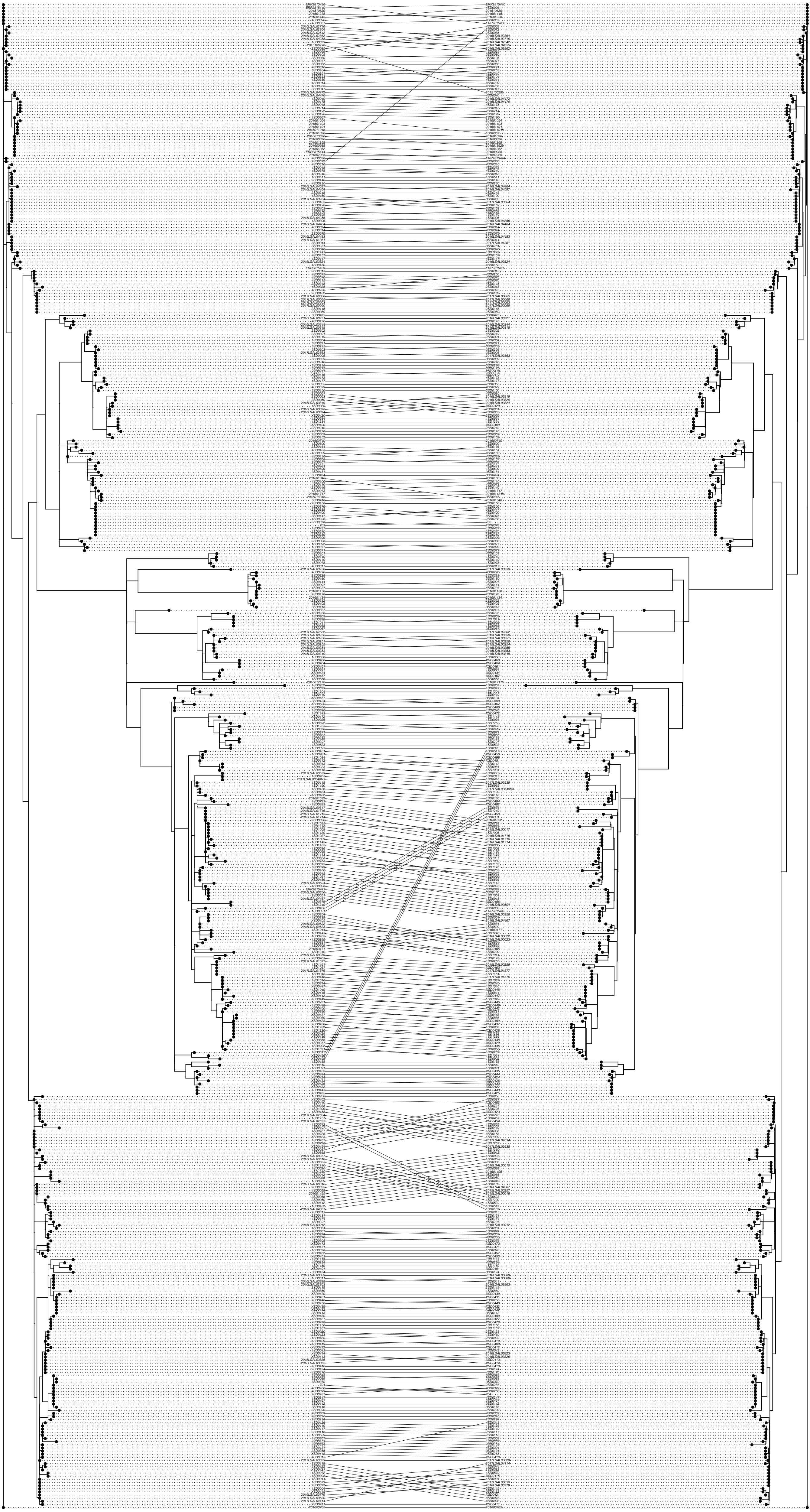

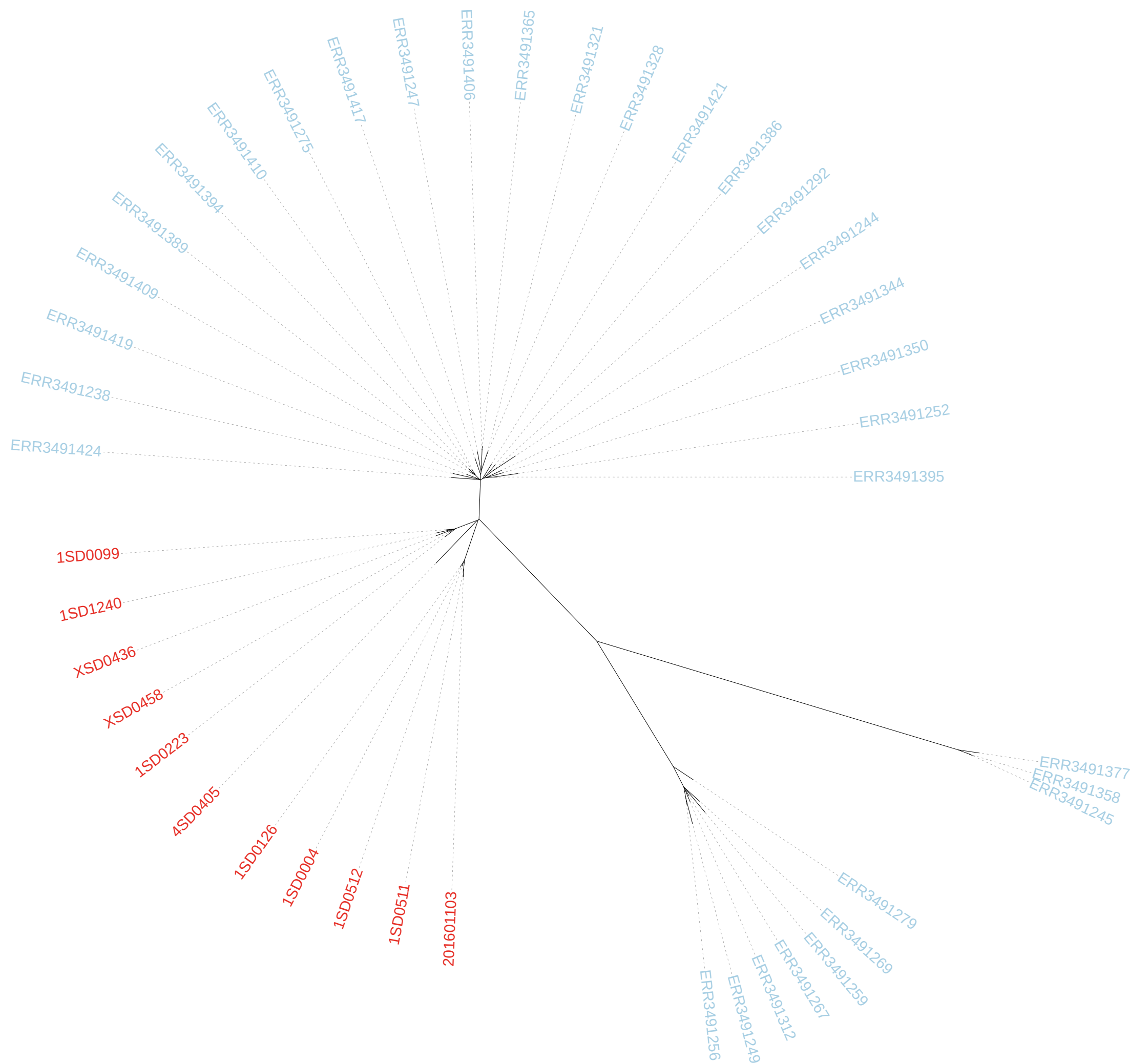

Clusters

61  
56  
51  
46  
41  
36  
31  
26  
21  
16  
11  
6  
1

1 6 11 16 21 26 31 36 41 46 51 56 61

Clusters

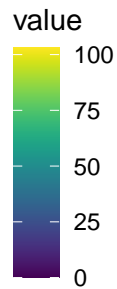

A horizontal number line with a single tick mark in the middle.

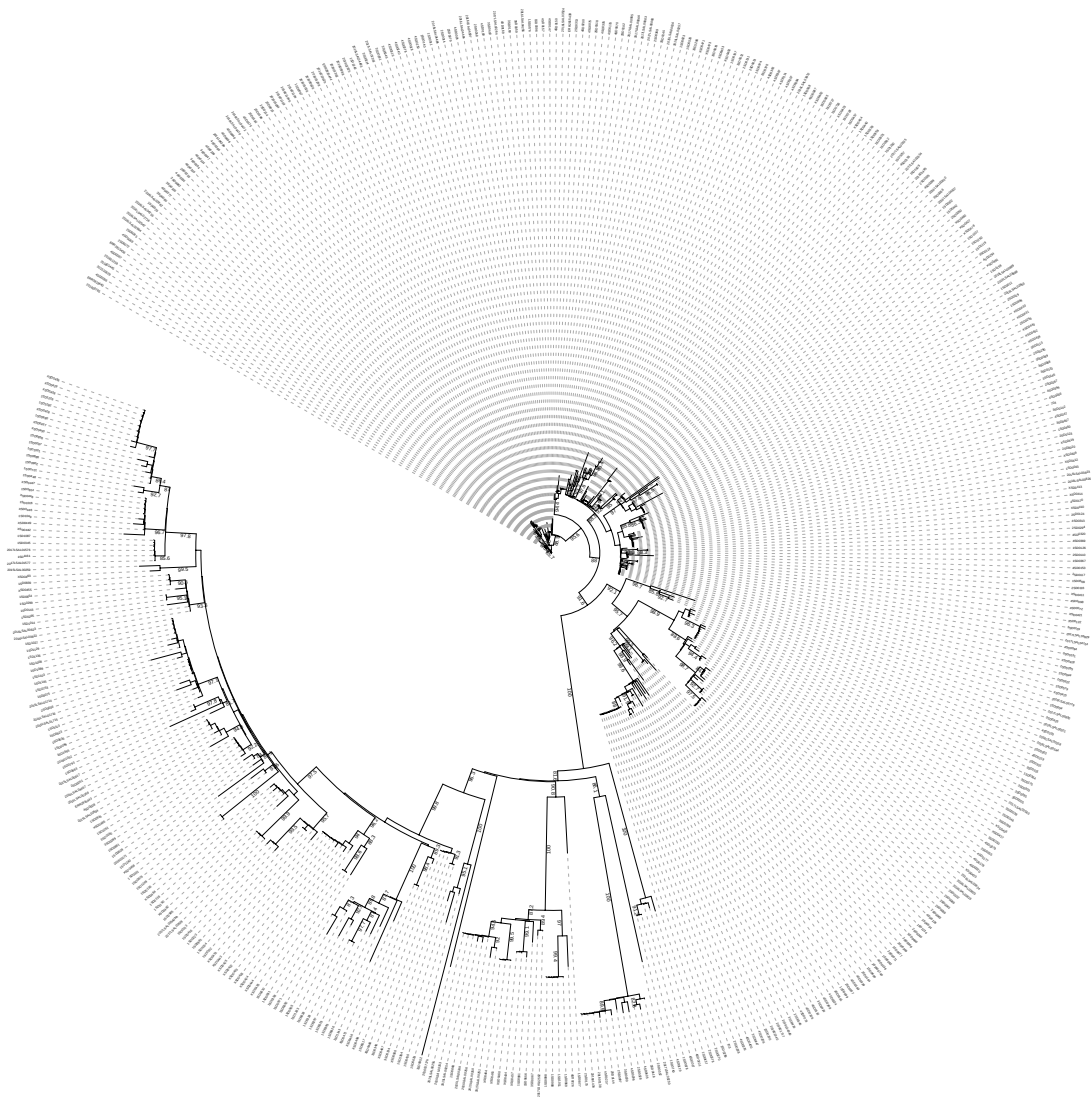

Supplement: lqac047_Supplemental_Files [file lqac047_supplemental_files.zip › supplementary_data.pdf]
